# Efficacy of Levofloxacin Based Triple and High-Dose PPI-Amoxicillin Dual Eradication Therapy for *Helicobacter pylori* after Failures of First- and Second-Line Therapies

**DOI:** 10.1155/2014/631501

**Published:** 2014-12-16

**Authors:** Kenichiro Okimoto, Makoto Arai, Keiko Saito, Shoko Minemura, Daisuke Maruoka, Tomoaki Matsumura, Tomoo Nakagawa, Tatsuro Katsuno, Chisato Ishii, Shota Murata, Masaharu Watanabe, Fumio Nomura, Osamu Yokosuka

**Affiliations:** ^1^Department of Gastroenterology and Nephrology, Graduate School of Medicine, Chiba University, Inohana 1-8-1, Chiba City 260-8670, Japan; ^2^Department of Clinical Laboratory, Chiba University Hospital, Japan

## Abstract

*Objectives*. The aim of this study was to investigate and compare the eradication rate of *Helicobacter pylori* as the third-line triple therapy with rabeprazole (RPZ) + amoxicillin (AMPC) + levofloxacin (LVFX) and high-dose RPZ + AMPC. *Methods*. 51 patients who failed Japanese first-line (proton pump inhibitor (PPI) + AMPC + clarithromycin) and second-line (PPI + AMPC + metronidazole) eradication therapy were randomly assigned at a 1 : 1 ratio to one of the following third-line eradication groups: (1) RAL group: RPZ 10 mg (b.i.d.), AMPC 750 mg (b.i.d.), and LVFX 500 mg (o.d.) for 10 days; (2) RA group: RPZ 10 mg (q.i.d.) and AMPC 500 mg (q.i.d.) for 14 days. Patients who failed to respond to third-line eradication therapy received salvage therapy. *Results*. The rates of eradication success, based on intention to treat (ITT) analysis, were 45.8% in the RAL group and 40.7% in the RA group. The overall eradication rates were 73.9% in the RAL group and 64.0% in the RA group. There was no significant difference between the two groups. *Conclusions*. The third-line triple therapy with RPZ, AMPC, and LVFX was as effective as that with high-dose RPZ and AMPC.

## 1. Introduction

*Helicobacter pylori* (*H. pylori*) is a Gram-negative bacillus that inhabits the gastric mucosa and mucus.* H. pylori* infection is now known to be a risk factor of a wide range of diseases, such as atrophic gastritis, gastroduodenal ulcer, mucosa-associated lymphoid tissue (MALT) lymphoma, gastric cancer, and idiopathic thrombocytopenic purpura (ITP) [1–4]. Furthermore, the recurrence of early gastric cancer after its endoscopic resection was reported to be reduced to almost one-third if* H. pylori* was successfully eradicated [5]. In Japan 150,000 deaths from gastric cancer will be prevented over 5 years if all* H. pylori* is eliminated [6].

The first-line eradication therapy of* H. pylori* in Japan is triple therapy, consisting of a proton pump inhibitor (PPI), amoxicillin (AMPC), and clarithromycin (CAM). This regimen has been covered by the national health insurance system since December 2000. However, due to the increase of CAM-resistant* H. pylori* strains, the eradication rate of first-line therapy is reported to be as low as 75% [7]. Second-line eradication therapy, consisting of PPI, AMPC, and metronidazole (MNZ), has been approved in Japan for those who fail the first-line therapy. The eradication rate of the second-line therapy has been reported to be around 90% [8, 9]. These results indicate that about 3% of* H. pylori* positive patients fail in both first- and second-line therapy. From 2013, the eradication treatment for* H. pylori* infection with chronic gastritis was covered by the national health insurance in Japan. As the number of the patients who are eligible for the eradication of* H. pylori* is estimated to be increasing, it is becoming more important to establish standard third-line eradication therapy. The guidelines of the Japanese Society of Helicobacter Research suggest PPI + AMPC + levofloxacin (LVFX) or high-dose PPI + AMPC as the third-line eradication regimen in 2009. In the former regimen, the rate of eradication was reported to be 43.1–70.0% if it was used as third-line therapy [10, 11]. In the latter regimen, the rate of eradication was reported to be 54.3% if it was used as third-line therapy [11]. Until now, which regimen is more effective for third-line eradication therapy has not been clarified sufficiently. The aim of this study was to investigate and compare the eradication rate of third-line triple therapy with rabeprazole (RPZ) + AMPC + LVFX to high-dose RPZ + AMPC which were suggested in the guidelines of the Japanese Society of Helicobacter Research in 2009.

## 2. Methods

### 2.1. Study Design and Patients

This was a prospective, randomized, controlled study conducted in Chiba University Hospital. From June 2011 to June 2013, patients who failed Japanese first-line (PPI + AMPC + CAM) and second-line (PPI + AMPC + MNZ) eradication therapy were randomly assigned to one of the following third-line eradication groups at a 1 : 1 ratio using random number tables: (1) RAL group: RPZ 10 mg (b.i.d.), AMPC 750 mg (b.i.d.), and LVFX 500 mg (o.d.) for 10 days; (2) RA group: RPZ 10 mg (q.i.d.) and AMPC 500 mg (q.i.d.) for 14 days. Patients (1) who had a past history of allergy to the study drugs, (2) who had severe liver dysfunction, (3) who were undergoing treatment of malignant disease, (4) who were pregnant or lactating, or (5) who were ineligible to participate in this study according to the decision of a physician were excluded from this study. The primary endpoint was to compare the eradication rate between RAL and RA group. The secondary endpoints were the safety of treatment and the efficacy of fourth-line treatment.

Before being randomized, all patients underwent upper endoscopy and we performed* H. pylori* sensitivity testing for the antibiotics (AMPC, CAM, MNZ, and LVFX). Six to twelve weeks after the end of the third-line eradication therapy, a ^13^C-urea breath test (UBT) was performed. Patients who failed to respond to third-line eradication therapy received salvage therapy as the fourth-line one. In this regimen, patients who were treated with RAL received RA (salvage RA group) and those treated with RA received RAL (savage RAL group). Six to twelve weeks after the end of the salvage therapy,* H. pylori* eradication was assessed again with UBT. During the study, blood samples were taken from the patients to analyze the cytochrome P450 2C19 (CYP2C19) polymorphism.

This study was conducted according to the principles of the Declaration of Helsinki. All participating patients gave written informed consent. The study protocol was approved by Chiba University Institutional Review Board and registered (Clinical Registration Number, UMIN000005373).

### 2.2. Assessment of the Presence and Eradication of* H. pylori*

All patients underwent UBT before third-line eradication to check for the presence of* H. pylori*. At 6–12 weeks after the third-line therapy and salvage therapy, the patients whose UBT results were negative were considered to have eradicated the infection. Before UBT, PPI, which may influence the test results, was discontinued for at least 2 weeks.

### 2.3. *H. pylori* Testing for Sensitivity to the Antibiotics

*H. pylori* strains were isolated from tissue samples from the gastric corpus and antibiotic sensitivity testing was performed using the *E*-test. The breakpoints were 1.0 *μ*g/mL for ABPC, CAM, and LVFX and 8.0 *μ*g/mL for MNZ. An* H. pylori* strain was judged resistant to the antibiotics when its minimum inhibitory concentrations (MIC) value was equal to or beyond the breakpoint.

### 2.4. CYP2C19 Gene Polymorphism

CYP2C19 gene polymorphism was analyzed. Genotyping of the two mutated genes was performed using the gene analysis by fluorescence correlation spectroscopy (gFCS) method with blood serum samples from the patients. The CYP2C19 gene polymorphisms were classified into three types: CYP2C19 wild type gene (CYP2C19^∗^1) and two mutated genes (CYP2C19^∗^2 and CYP2C19^∗^3). Patients with (^∗^1/^∗^1) were categorized as the homogeneous extensive metabolizers (EM). Patients with (^∗^1/^∗^2) or (^∗^1/^∗^3) were categorized as the heterogeneous EM. And patients with (^∗^2/^∗^3), (^∗^2/^∗^2), or (^∗^3/^∗^3) were categorized as the poor metabolizers (PM).

### 2.5. Adverse Events

Adverse events (AE) were evaluated by asking the patients about their condition and laboratory evaluation of liver and renal function, which was performed two weeks after the end of the third-line eradication therapy.

### 2.6. Statistical Analysis

Statistical analysis of the third and overall eradiation rates was performed between two groups using the chi-square test. Each parameter of the patient background was analyzed using the chi-square test or unpaired *t*-test. Adverse events were analyzed using the chi-square test. A *P* value of <0.05 was considered to be statistically significant. Statistical analysis was performed by the software SPSS 16.0J (SPSS Inc., Chicago, IL, USA), with *P* < 0.05 considered statistically significant.

## 3. Results

### 3.1. Study Patients

**Figure 1:**
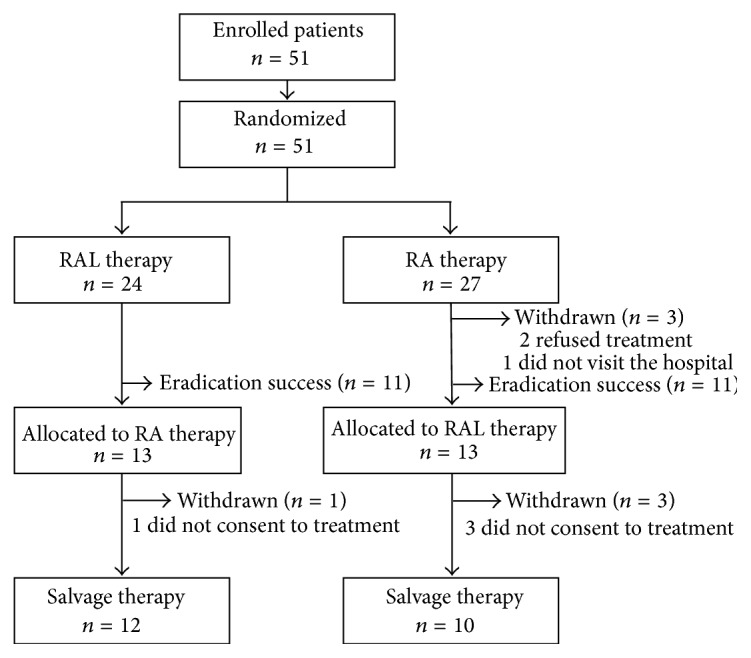
Flow diagram of the study. Enrolled patients were randomly assigned to either the RAL group, RPZ 10 mg (b.i.d.), AMPC 750 mg (b.i.d.), and LVFX 500 mg (o.d.) for 10 days, or the RA group, RPZ 10 mg (q.i.d.) and AMPC 500 mg (q.i.d). Patients who failed to respond to third-line eradication therapy underwent salvage therapy.

Fifty-one patients were enrolled in this study and the study flow of the patients is shown in Figure 1. The 51 patients were randomly assigned to the RAL group (*n* = 24) or RA group (*n* = 27) (intension to treat (ITT) analysis subjects set). Three patients in the RA group were excluded from the per-protocol (PP) analysis because two refused treatment and one did not visit the hospital. As a result, 24 patients in each group were included in the PP analysis.* H. pylori* was eradicated from eleven patients in each group. Twelve patients in the RAL group and eleven patients in the RA group underwent salvage therapy. One patient in the RAL group and two patients in the RA group did not consent to salvage therapy. One patient in the salvage RAL group who refused treatment was excluded from PP analysis. Consequently, twelve patients in the RAL group and ten patients in the RA group were included in the PP analysis.* H. pylori* was eradicated from six patients in the RAL group (salvage RA group) and five patients in the RA group (salvage RAL group).

### 3.2. Backgrounds of the Patients

The backgrounds of the patients, based on PP analysis, are shown in Table 1. The two groups had similar characteristics with respect to age, sex, body mass index (BMI), reasons for the eradication, and success rate of the drug sensitivity test. The success rate of the drug sensitivity testing was defined as the ratio of number of cases with them to all cases. Four patients refused the analysis of CYP2C19. The number of CYP2C19 EM was significantly higher in the RAL group than in the RA group (*P* < 0.05).

### 3.3. Success Rate of the Third-Line Eradication Therapy

Drug compliance exceeded 90% for all patients included in PP analysis. The rates of eradication, based on ITT analysis, were 45.8% for the RAL group and 40.7% for the RA group and there was no significant difference (*P* = 0.71). In the PP analysis, the rates of eradication were the same (45.8%) in both groups.

### 3.4. Comparison of the Patients' Backgrounds between the Success and Failure Groups in the Third-Line Eradication Therapy

Table 2 shows a comparison, based on PP analysis, of the patients' backgrounds, between the success and failure groups in the third-line eradication therapy. There was no difference with respect to sex, BMI, eradication therapy, drug resistance, and CYP2C19 polymorphism. The age (mean ± S.D.) of the success group was 64.1 ± 10.0, which was significantly higher than that of the failure group (56.2 ± 13.5, *P* < 0.05). The success rate of the drug sensitivity test was significantly higher for the failure group than for the success group (80.0% versus 36.4%, *P* < 0.05).

### 3.5. Salvage Therapy and Overall Eradication Rates

**Figure 2:**
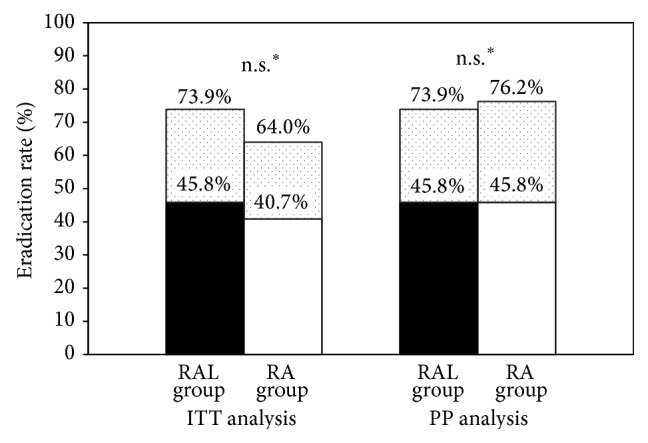
ITT and PP analysis of the overall success rate (third-line therapy and salvage therapy eradication rate). Black and white areas indicate the success rates of third-line eradication therapy. The dotted area indicates the additional success rate of the following salvage therapy. ^∗^Chi-square test.

The rates of eradication success in salvage eradication therapy were 50.0% in the RAL group (salvage RA group) and 45.5% in the RA group (salvage RAL group), based on ITT analysis. There was no significant difference (*P* = 0.83). In PP analysis, the eradication rate was 50% in both groups. The overall eradication rate (third-line and salvage therapy eradication rate) was 73.9% in the RAL group and 64.0% in the RA group, based on ITT analysis (*P* = 0.46). In PP analysis, the overall eradication rate was 73.9% in the RAL group and 76.2% in the RA group (*P* = 0.86), which showed no significant difference (chi-square test, Figure 2).

### 3.6. Adverse Events

AE of the third-line eradication therapy is shown in Table 3. In both groups, the most frequently observed AE was soft stool/diarrhea (20.8% in both groups). A rash was observed in one patient (4.2%) in the RAL group and one patient in the RA group (4.2%) had nausea. There was no significant difference between the two groups regarding any AE and all AE were reversible.

## 4. Discussion

Recently, sitafloxacin- (STFX-) based third-line eradication therapy has been reported to be effective, with an eradication rate of 70–83.6% [11–13]. The reasons for the relatively high eradication rate might be attributable to the following factors. The DNA gyrase subunit A (*gry*A) mutation is known to be responsible for the resistance of* H. pylori* to quinolones [14]. It was reported that the MIC (50) (MIC for 50% of the organisms) of STFX against gyrA mutants is 0.12 *μ*g/mL, significantly lower than that of LVFX (8 *μ*g/mL) [12]. Furthermore, the* H. pylori* resistance rate to STFX was 7.7%, which was lower than that of LVFX [11]. Although STFX has some benefits, it is available only in Japan and Thailand now. Taking this current state into consideration, LVFX-based triple therapy and high-dose PPI and AMPC dual therapy may be widely available in many countries, as well as in Japan. In a previous study [11], STFX-based third-line therapy was superior to that based on LVFX but the regimen requires patients to take LVFX 300 mg (b.i.d). In our LVFX-based triple therapy, the patients take LVFX 500 mg (o.d.). Because LVFX is dependent on concentration, our regimen makes the best use of the drug characteristics. In this study, the eradication rate of the RAL group was 45.8% on ITT analysis, which was almost the same as the RA group (40.7%). The third-line RAL or RA may be inferior to STFX-based therapy.

In this study, LVFX and AMPC were selected as the antibiotics for the third-line and salvage eradication therapy. Currently, LVFX is used for a wide range of infectious diseases and is the most frequently used quinolone for* H. pylori* eradication [12]. As a result, a high percentage of* H. pylori* strains are LVFX-resistant. The resistance rate of* H. pylori* to LVFX has been reported to be 39–57% [11, 15]. In this study it was 46.4%, similar to previous reports. This might have influenced the relatively low eradication rate of the RAL group. On the other hand, the resistance rate of* H. pylori* to AMPC has been reported to be low in Japan, the United States, Europe, and China [16]. Consequently the eradication rate of a high-dose PPI + AMPC regimen has been reported to be 90.9 to 100% when used as second-line eradication therapy [17, 18]. Contrary to previous studies, Murakami et al. reported that the resistance rate of* H. pylori* to AMPC in patients who failed both first- and second-line AMPC-based eradication therapy was 8.2% and the eradication rate of a high-dose PPI + AMPC regimen as the third-line therapy was 54.3% [11]. The resistance rate was relatively high. It is possible that a proportion of* H. pylori* strains acquire resistance to AMPC during first- and second-line eradication therapy. In our study, although the rate of resistance of* H. pylori* to AMPC was 0%, 40.4% of the patients had failed the drug sensitivity test and, in some of these cases, AMPC-resistant* H. pylori* strains may have been present. This could be a cause of the low eradication rate (40.7% on ITT analysis).

Increased PPI dosing was reported to be critical for the success of* H. pylori* eradication [19]. PPI is generally metabolized in the liver by CYP2C19. In Japan, the percentages of the EM and PM genotypes of CYP2C19 are 89% and 11%, respectively [20]. Lee et al. reported that patients with the PM genotype of CYP2C19 achieved a high* H. pylori* eradication rate compared to those with EM [21]. Among the many PPIs, rabeprazole is mainly reduced to rabeprazole thioether by a nonenzymatic pathway and partially metabolized to demethylated rabeprazole by CYP2C19. Therefore, its metabolism is not influenced by the CYP2C19 polymorphism [22]. Rabeprazole was used in this study and the ratio of EM did not differ significantly between the success and failure groups in third-line eradication therapy.

This study has some limitations. Firstly, the number of study patients was small. Secondly, a comparison with STFX-based therapy was not implemented. In the future, a large scale prospective study, including other suggestive regimens, should be carried out to establish standard third-line eradication therapy for* H. pylori*. Thirdly, in spite of randomization, there exists some difference of the clinical background between RAL and RA groups. Particularly, CYP2C19 genotype showed the significant difference between RAL and RA groups. Because the success rate of* H. pylori* eradication with third-line therapy or salvage therapy did not show the difference between RAL and RA groups, we speculated that the influence of this uneven distribution might be low.

In conclusion, as the third-line triple therapy, the eradication rate with rabeprazole, AMPC, and LVFX for 10 days was equal to that with high-dose rabeprazole and AMPC for 14 days. These efficacy rates were not high but can be an alternative therapy after second-line failure because these therapies seemed free from severe adverse events.

## Figures and Tables

**Table 1:** Background of the patients based on PP analysis.

	RAL group^†^ (*n* = 24)	RA group^‡^ (*n* = 24)	*P* value
Age (mean ± S.D.)	57.8 ± 12.6	61.8 ± 12.6	n.s.^∗^
Sex (male/female)	10/14	8/16	n.s.^∗∗^
BMI^§^ (mean ± S.D.)	23.0 ± 3.7	21.5 ± 3.4	n.s.^∗^
Disease, *n* (%)			
Gastric ulcer	5 (20.8)	5 (20.8)	n.s.^∗∗^
Duodenal ulcer	3 (12.5)	5 (20.8)	n.s.^∗∗^
Early gastric cancer	4 (16.7)	3 (12.5)	n.s.^∗∗^
Hyperplastic polyp	2 (8.3)	0 (0)	n.s.^∗∗^
Others	10 (41.7)	11 (45.8)	n.s.^∗∗^
Success of *H. pylori* culture (yes/no)^a^	17/7	11/12	n.s.^∗∗^
Drug resistance, *n* (%)			
Amoxicillin	0 (0)	0 (0)	n.s.^∗∗^
Clarithromycin	15 (88.2)	8 (72.7)	n.s.^∗∗^
Metronidazole	13 (76.5)	9 (81.8)	n.s.^∗∗^
Levofloxacin	6 (35.3)	7 (63.6)	n.s.^∗∗^
CYP2C19 polymorphism (EM^||^/PM^¶^)^b^	20/2	13/9	<0.05^∗∗^

^†^RAL group: RPZ 10 mg (b.i.d.), AMPC 750 mg (b.i.d.), and LVFX 500 mg (o.d.).

^‡^RA group: RPZ 10 mg (q.i.d.) and AMPC 500 mg (q.i.d.).

^§^BMI, body mass index; ^||^EM, extensive metabolizer; ^¶^PM, poor metabolizer; ^a^one patient in RA group refused the drug sensitivity test; ^b^four patients (two in RAL group and two in RA group) refused the analysis of CYP2C19 polymorphism; ^∗^unpaired *t*-test; ^∗∗^chi-square test.

**Table 2:** Comparison of the patients' backgrounds between the success and failure groups, based on PP analysis (third-line eradication therapy).

	Success group (*n* = 22)	Failure group (*n* = 26)	*P* value
Age (mean ± S.D.)	64.1 ± 10.0	56.2 ± 13.5	<0.05^∗^
Sex (male/female)	11/11	7/19	n.s.^∗∗^
BMI^†^ (mean ± S.D.)	22.0 ± 3.3	22.3 ± 4.0	n.s.^∗^
Eradication therapy (RAL/RA)	11/11	13/13	n.s.^∗∗^
Success of *H. pylori* culture (yes/no)^a^	8/14	20/5	<0.05^∗∗^
Drug resistance, *n* (%)			
Amoxicillin	0 (0)	0 (0)	n.s.^∗∗^
Clarithromycin	6 (75.0)	17 (81.0)	n.s.^∗∗^
Metronidazole	6 (75.0)	16 (76.2)	n.s.^∗∗^
Levofloxacin	3 (37.5)	10 (47.6)	n.s.^∗∗^
CYP2C19 polymorphism (EM^‡^/PM^§^)^b^	14/5	19/6	n.s.^∗∗^

^†^BMI, body mass index; ^‡^EM, extensive metabolizer; ^§^PM, poor metabolizer; ^a^one patient in failure group refused the drug sensitivity test; ^b^four patients (three in success group and one in failure group) refused the analysis of CYP2C19 polymorphism; ^∗^unpaired *t*-test; ^∗∗^chi-square test.

**Table 3:** Adverse events of the third-line eradication therapy.

	RAL group^†^ (*n* = 24)	RA group^ ‡^ (*n* = 24)	*P* value
Soft stool/diarrhea, *n* (%)	5 (20.8)	5 (20.8)	n.s.^∗^
Nausea, *n* (%)	0 (0)	1 (4.2)	n.s.^∗^
Rash, *n* (%)	1 (4.2)	0 (0)	n.s.^∗^

^†^RAL group: RPZ 10 mg (b.i.d.), AMPC 750 mg (b.i.d.), and LVFX 500 mg (o.d.).

^‡^RA group: RPZ 10 mg (q.i.d.) and AMPC 500 mg (q.i.d.).

^∗^Chi-square test.

## Abbreviations

MALT: Mucosa-associated lymphoid tissue
ITP: Idiopathic thrombocytopenic purpura
PPI: Proton pump inhibitor
AMPC: Amoxicillin
CAM: Clarithromycin
MNZ: Metronidazole
LVFX: Levofloxacin
RPZ: Rabeprazole
UBT: ^13^C-urea breath test
CYP2C19: Cytochrome P450 2C19
MIC: Minimum inhibitory concentrations
gFCS: Gene analysis by fluorescence correlation spectroscopy
EM: Extensive metabolizer
PM: Poor metabolizer
AE: Adverse event
ITT: Intension to treat
PP: Per-protocol
BMI: Body mass index
STFX: Sitafloxacin
*gry*A: DNA gyrase subunit A.
